# Activation of Nlrp3 Inflammasomes Enhances Macrophage Lipid-Deposition and Migration: Implication of a Novel Role of Inflammasome in Atherogenesis

**DOI:** 10.1371/journal.pone.0087552

**Published:** 2014-01-27

**Authors:** Xiang Li, Yang Zhang, Min Xia, Erich Gulbins, Krishna M. Boini, Pin-Lan Li

**Affiliations:** 1 Department of Pharmacology and Toxicology, School of Medicine, Virginia Commonwealth University, Richmond, Virginia, United States of America; 2 Department of Molecular Biology, Medical School Essen, University of Duisburg-Essen, Essen, Germany; University of California Merced, United States of America

## Abstract

Although Nlrp3 inflammasome activation in macrophages has been shown to be critical for the development of atherosclerosis upon atherogenic stimuli, it remains unknown whether activated Nlrp3 inflammasomes by other non-atherogenic stimuli induce alterations in macrophages that may contribute in the concert with other factors to atherogenesis. Thus, the present study tested the hypothesis that activation of Nlrp3 inflammasomes by ATP, which is a classical non-lipid danger stimulus, enhances the migration of macrophage and increases lipids deposition in macrophages accelerating foam cell formation. We first demonstrated that extracellular ATP (2.5 mM) markedly increased the formation and activation of Nlrp3 inflammasomes in bone marrow macrophages (BMMs) from wild type (Asc^+/+^) mice resulting in activation of caspase-1 and IL-1β production. In these Asc^+/+^ macrophages, such stimulation of inflammasomes by non-lipid ATP was similar to those induced by atherogenic stimuli such as cholesterol crystals or 7-ketocholesterol. Both non-lipid and lipid forms of stimuli induced formation and activation of Nlrp3 inflammasomes, which were prevented by Asc gene deletion. Interestingly, Asc^+/+^ BMMs had dramatic lipids accumulation after stimulation with ATP. Further, we demonstrated that large amount of cholesterol was accumulated in lysosomes of Asc^+/+^ BMMs when inflammasomes were activated by ATP. Such intracellular and lysosomal lipids deposition was not observed in Asc^−/−^ BMMs and also prevented by caspase-1 inhibitor WEHD. In addition, *in vitro* and *in vivo* experiments revealed that migration of Asc^+/+^ BMMs increased due to stimulation of Nlrp3 inflammasomes, which was markedly attenuated in Asc^−/−^ BMMs. Together, these results suggest that activation of Nlrp3 inflammasomes remarkably increases the susceptibility of macrophages to lipid deposition and their migration ability. Such novel action of inflammasomes may facilitate entry or retention of macrophages into the arterial wall, where they form foam cells and ultimately induce atherosclerosis.

## Introduction

The inflammasome is intracellular inflammatory machinery that has been reported to switch on the inflammatory response of tissues or organs to various danger signals [1], [2]. Among different types of inflammasomes, the Nod-like receptor family pyrin domain containing 3 (Nlrp3) inflammasome is well characterized in a variety of mammalian cells, as a receptor for endogenous danger signals such as ATP, cholesterol crystals, β-amyloid and monosodium urate [3], [4], [5], [6], [7], [8], [9]. The Nlrp3 inflammasome typically forms a complex with the adaptor protein apoptosis-associated speck-like protein (Asc) and caspase-1. Stimulation with danger or inflammatory signals triggers Nlrp3 inflammasome assembly and the formation of a large multi-molecular complex that controls caspase-1 activity and subsequent bioactive IL-1β production [4], [10], [11], [12], [13].

There is substantial evidence showing that accumulated lipids in macrophages exist not only in cytoplasmic inclusions as cholesteryl ester inclusions but also in lipid-swollen lysosomes as both free cholesterol and cholesteryl ester during atherosclerosis [14], [15]. This lipid deposition in the cytosol and lysosomes critically contributes to the foam cell formation in arterial walls, ultimately resulting in arterial wall fibrosis or atherosclerosis [16]. It is known that the foam cells are important to the formation of atherosclerotic plaques and ultimate arterial sclerosis and that vascular inflammation may initiate such pathological changes in macrophages. However, it is unknown whether activation of inflammasomes in macrophages even before vascular inflammation also contributes to atherosclerosis. In particular, it is imperative to know whether non-lipid danger signals activating the inflammasome in macrophages may alter lipid metabolism or transport in these cells and thereby increase the potential to lead to the formation of foam cells and sclerosis in arterial walls. This is particular relevant to the development of atherosclerosis in patients with chronic inflammation or with chronic or remittent bacterial or viral infections, where they may have activated inflammasomes through damage-associated molecular patterns (DAMPs) or pathogen-associated molecular patterns (PAMPs).

The present study was designed to test the hypothesis that activation of Nlrp3 inflammasomes even by non-lipid danger stimuli such as ATP impairs the ability of macrophages to properly handle lipid metabolism or transport and enhances their migration capacity to accelerate foam cell formation and migration to inflammatory tissues. Our data show a formation and activation of Nlrp3 inflammasomes in bone marrow derived macrophages (BMMs) in response to ATP (non-lipid), cholesterol crystal and 7-ketocholestrol (lipid). ATP-induced activation of Nlrp3 inflammasomes caused a deposition of cholesterol in lysosomes in BMMs and enhanced migration of these cells, events that were abrogated or attenuated by Asc gene knockout. Our findings support the view that activation of Nlrp3 inflammasomes in macrophages even by non-lipid stimuli may alter macrophage's lipid metabolism and migration, thereby increasing the susceptibility of foam cell formation and macrophage infiltration into local inflammatory tissues. During atherogenesis, such actions of Nlrp3 inflammasomes are distinct from typical inflammasome-secreted cytokine (IL-1β/IL18)-induced inflammatory responses such as T-cell infiltration in the vasculature, which may implicate an uncanonical non-inflammatory role of inflammasomes in atherogenesis.

## Materials and Methods

### Mice

Asc^−/−^ and wild-type mice (Asc^+/+^) have been described previously [6]. Mice were housed in a pathogen free facility. Male and female mice, 8 weeks of age, were used in all experiments. Then genotype of Asc^−/−^ mice was confirmed by RT-PCR and by the absence of IL-1β release to monosodium urate crystals (MSU, Invivogen). All protocols were approved by the Institutional Animal Care and Use Committee of the Virginia Commonwealth University.

### Primary culture of bone marrow-derived macrophages (BMMs)

Bone marrow macrophages (BMMs) were cultured as we described previously [17]. Briefly, bone marrow cells were harvested by flushing mouse femurs and tibias with 4 mL BMM medium: DMEM Gluta MAX with sodium pyruvate medium supplemented with 10% fetal bovine serum (GIBCO BRL), 10 mM HEPES (Fisher Scientific), 1% NEAA (GIBCO BRL), 100 U of penicillin per ml, 100 µg of streptomycin (BioWhittaker) per ml, and 15% L-cell supernatant as a source of macrophage colony-stimulating factor. The cells were passed through a 23G needle in a 10 ml syringe for 5 times and seeded in T25 plate. After 4 days in culture, macrophages were supplied with fresh BMM media, and mature macrophages were used on day 7 of culture. BMMs were collected by treating cells with 0.25% trypsin/EDTA. BMMs (5×10^4^) were cultured in 1.0 ml fresh BMM media as described above in a 12-well plate or as indicated. BMMs were primed with low dose of LPS (1 ng/ml) for 3 h before any experiments. Cholesterol crystals were prepared as described previously [8]. ATP and 7-ketocholesterol were purchased from Sigma and freshly prepared before experiments. IL1β, IL1R antagonist and IL18 were purchased from Sigma.

### Confocal microscopy analysis

For confocal analysis of inflammasome molecules in BMMs, cultured cells were grown on glass coverslips, stimulated or unstimulated, fixed in 4% paraformaldehyde in phosphate-buffer saline (PFA/PBS) for 15 min. After being permeablized with 0.1% Triton X-100/PBS and rinsed with PBS, the cells were blocked with 3% donkey serum, and then incubated 1 h at room temperature with goat anti-Nlrp3 (1∶200, Abcam, MA) and rabbit anti-Asc (1∶100, Enzo, PA) or rabbit anti-caspase-1 (1∶100, Abcam, MA). After washing, these slides were probed with primary antibodies and incubated with Alexa-488- or Alexa-555-labeled secondary antibodies for 1 h at room temperature. The slides were mounted and subjected to examinations using a confocal laser scanning microscope (Fluoview FV1000, Olympus, Japan), with photos being taken and the colocalization of Nlrp3 with Asc or caspase-1 analyzed by the Image Pro Plus 6.0 software (Media Cybernetics, Bethesda, MD, USA). The summarized colocalization efficiency data was expressed as Pearson correlation coefficient (PCC) as we described previously [18].

### Western blot analysis

Protein from cell lysate was run on a SDS-PAGE gel, transferred into PVDF membrane and blocked. Then the membrane was probed with primary antibodies against active caspase-1 (1∶500 dilution; Santa Cruz Biotech.) overnight at 4°C followed by incubation with horseradish peroxidase-labeled IgG. The immunoreactive bands were detected by chemiluminescence methods and visualized on Kodak Omat film. β-actin (1∶500 dilution; Santa Cruz Biotech.) was reprobed to serve as a loading control. The intensity of the bands was quantified by densitometry using ImageJ software [19].

### IL-1β assay

The cell supernatant of each well (2×10^5^ BMMs) in a 12-well plate was collected to measure the IL-1β production by a mouse IL-1β ELISA kit (R&D systems, MN) according to the protocol described by the manufacturer.

### FLICA staining of active caspase-1

After stimulation, BMM were labeled with a green fluorescent probe, FAM-YVAD-fmk caspase-1 FLICA™ (Immunochemistry, Bloomington, IN), which specifically binds the activate form of caspase-1. Therefore, higher caspase-1 activity will exhibit increased the percentage of FLICA-positive cells. Flow cytometric analysis was performed according to the manufacturer's manual. In brief, BMMs were incubated with FLICA probes and propidium iodide (PI) at room temperature for 1 h. After two washes with PBS, FLICA and PI fluorescence were analyzed by flow cytometry with a Guava EasyCyte (Guava Technologies, Hayward, CA). The percentage of FLICA-positive cells was used to represent relative caspase-1 activity and was normalized to Asc^+/+^ control group for comparison.

### Lipid deposition in BMM by Oil red O staining

For oil red O staining, the slides with BMMs were proceeded and stained as described previously [20] with minor modifications. BMMs (10^4^ cells/well) cultured in 200 µl BMM media in chamber slides were treated as indicated and loaded with oxidized low density lipoprotein (oxLDL, 10 µg/mL; KALEN Biomedical) for 16 hours. The slides with BMMs were then stained with Oil red O (0.1% in isopropanol) for lipid accumulation. The Oil red O staining was examined by light microscopy and images were obtained by MetaMorph 6.0. The data was represented by the area percentage of each cell positive for Oil red O stain, which was calculated in Image Pro Plus 6.0 software. For each sample, at least 200 cells were analyzed and summarized Oil red O positive percentages were used for statistical analysis.

### Confocal microscopic detection of lysosomal free cholesterol

To detect free cholesterol in lysosomes, confocal microscopy was performed to analyze co-staining of filipin (as a marker to stain free cholesterol) and Lamp1 antibody (lysosome marker) according to published methods [21], [22]. In brief, after treatment as indicated and exposure to oxLDL, BMMs in the chamber slides were stained with filipin (50 µg/ml) and rat anti-mouse Lamp1 (1∶500 dilution, BD Biosciences) in PBS containing 1% normal goat serum. The secondary antibody for Lamp1 was Alexa 555-conjuated goat anti-rat IgG (1∶300 dilution). The slides were mounted and subjected to examinations using a confocal laser scanning microscope (Fluoview FV1000, Olympus, Japan). The purple color spots in the merged image represent the deposited free cholesterol in lysosomes. The colocalization of colocalization efficiency data were analyzed by the Image Pro Plus 6.0 software (Media Cybernetics, Bethesda, MD, USA) and expressed as Pearson correlation coefficient (PCC) as we described previously [18]. For each sample, at least 200 cells were analyzed and summarized PCC data were used for statistical analysis.

### Assay of lysosomal cholesterol and ganglioside GM1

The lysosomes of mouse BMMs cultured in a 12-well plate were isolated by a lysosome isolation kit (LYSISO1, Sigma) with differential centrifugation, followed by a density gradient centrifugation and Ca^2+^ precipitation following the manufacturer's manual as we described previously [23]. The cholesterol concentrations in lysosomes were determined using a standard fluorescence assay kit (EnzyChrom AF Cholesterol Assay Kit) as described previously [24], [25]. Cholesterol is oxidized to yield hydrogen peroxide in a reaction catalyzed by cholesterol oxidase. In the presence of horseradish peroxidase, hydrogen peroxide is able to oxidize non-fluorescent 10-acetyl-3, 7-dihydroxyphenoxazine into highly fluorescent resorufin. The fluorescent intensity was determined at excitation/emission of 485/530 nm using a fluorescent microplate reader (FLx800, BIO-TEK Instruments) and used to calculate cholesterol concentrations following the manufacturer's instruction. The lysosomal ganglioside GM1 was determined by dot blot analysis as described previously [26]. The lysosomes from 10^6^ BMMs were isolated as described above and resuspended in 1 ml methanol. PVDF membrane was rinsed with methanol and dried in air. Then 2 µl of lysosomes in methanol were loaded on PVDF membrane. The membrane was then washed in PBS twice and incubated with a specific ganglioside GM1 binding agent, cholera toxin subunit B conjugated HRP (Invitrogen, 1∶10000), for 30 min. The ganglioside GM1 dots were detected by chemiluminescence methods and visualized on Kodak Omat film. The intensity of the bands was quantified by densitometry using ImageJ software.

### Confocal microscopic analysis of BODIPY-LacCer delivery to lysosomes

To show whether Nlrp3 inflammasome activation alters lipid transport in BMMs, the intracellular lipid trafficking was determined using BSA-conjugated BODIPY FL-C_5_-lactosylceramide (LacCer) and monitoring its endocytic trafficking as described previously [23], [27]. BMMs cultured in chamber slides were stimulated as indicated and then loaded with LacCer on ice for 15-min. To visualize the lysosomes, the cells were also labeled with 1 µM LysoTracker. After a wash with PBS, the PM bounded LacCer was back exchanged with ice-cold DMEM containing 5% fatty acid-free BSA at 4°C (3×15 min). Then BMMs were incubated at 37°C for a 30-min chase phase. At the end of chase point, the cells were subjected to examinations using a confocal laser scanning microscope (Fluoview FV1000, Olympus, Japan). The yellow spots in the merged image represent the delivery of LacCer from endosomes to lysosomes. The data was represented by the percentage of yellow spots in red spots analyzed in mage Pro Plus.

### Nucleofection of GFP plasmids

Transfection of plasmids encoding GFP (Lonza) into BMMs was performed using a 4D Nucleofector X-Unit (Lonza) according to the manufacturer's instructions as we described recently [28]. Briefly, BMMs were trypsinized and centrifuged at 90× g for 10 minutes. The cell pellet (2×10^6^ cells) was re-suspended in 100 µL P2 Nucleofection solution (Lonza) for Nucleofection with the program code DL100. For each Nucleofection sample, 2 µg GFP encoding plasmid DNA was added in 100 µL P2 Nucleofection solution. After Nucleofection, BMMs were cultured in the BMM media for 24 h and then used for the *in vivo* migration assay. The BMM viability post Nucleofection was determined by PI staining (0.5 µg/ml) using flow cytometric analysis (Guava Technologies, Hayward, CA).

### *In vitro* and *in vivo* migration assay

Migration of mouse BMMs was measured *in vitro* in a modified Boyden chamber migration assay using Transwell inserts with a 5 µm porous membrane (Corning) as described previously [29]. The BMMs (1.0×10^4^) were loaded into upper chamber of a 96-well transwell insert (Corning Inc.) with various treatments in the medium of the same upper chamber. Medium containing 10 ng/ml MCP-1 was placed in the lower chamber in some studies as positive control. After 24 h, the inserts were removed and the migratory BMMs in the lower chamber were counted. *In vivo* mouse macrophage migration was determined using a peritonitis model as described previously [30]. Briefly, GFP-expressing Asc^+/+^ or Asc^−/−^ BMMs were obtained by transfecting BMMs with GFP plasmids using high efficient Nucleofection technology as described above. Then, Asc^+/+^ mice were anesthetized with isoflurane and intravenously injected with GFP-expressing Asc^+/+^ or Asc^−/−^ BMMs (10^5^ cells per mouse). Migration of macrophages into peritoneal cavity was induced by injection of 1 ml of zymosan A (1 mg/ml) into the peritoneum. After 2 h, the mice were killed and the peritoneal lavages were rapidly collected for enumeration of leukocytes. The macrophages in the lavage fluids were detected by flow cytometry using APC-conjugated anti–F4/80 antibody, a macrophage marker (A&D Serotec Inc.). The exogenous GFP-expressing Asc^+/+^ or Asc^−/−^ BMMs were counted by analyzing cells that were double positive for GFP and APC-F4/80.

### Statistics

Data are presented as means ± SE. Significant differences between and within multiple groups were examined using ANOVA for repeated measures, followed by Bonferroni's multiple comparison tests. The Students *t* test was used to detect significant differences between two groups. P<0.05 was considered statistically significant.

## Results

### Induction of Nlrp3 inflammasome formation and activation by non-atherogenic or atherogenic stimuli in BMMs

**Figure 1:**
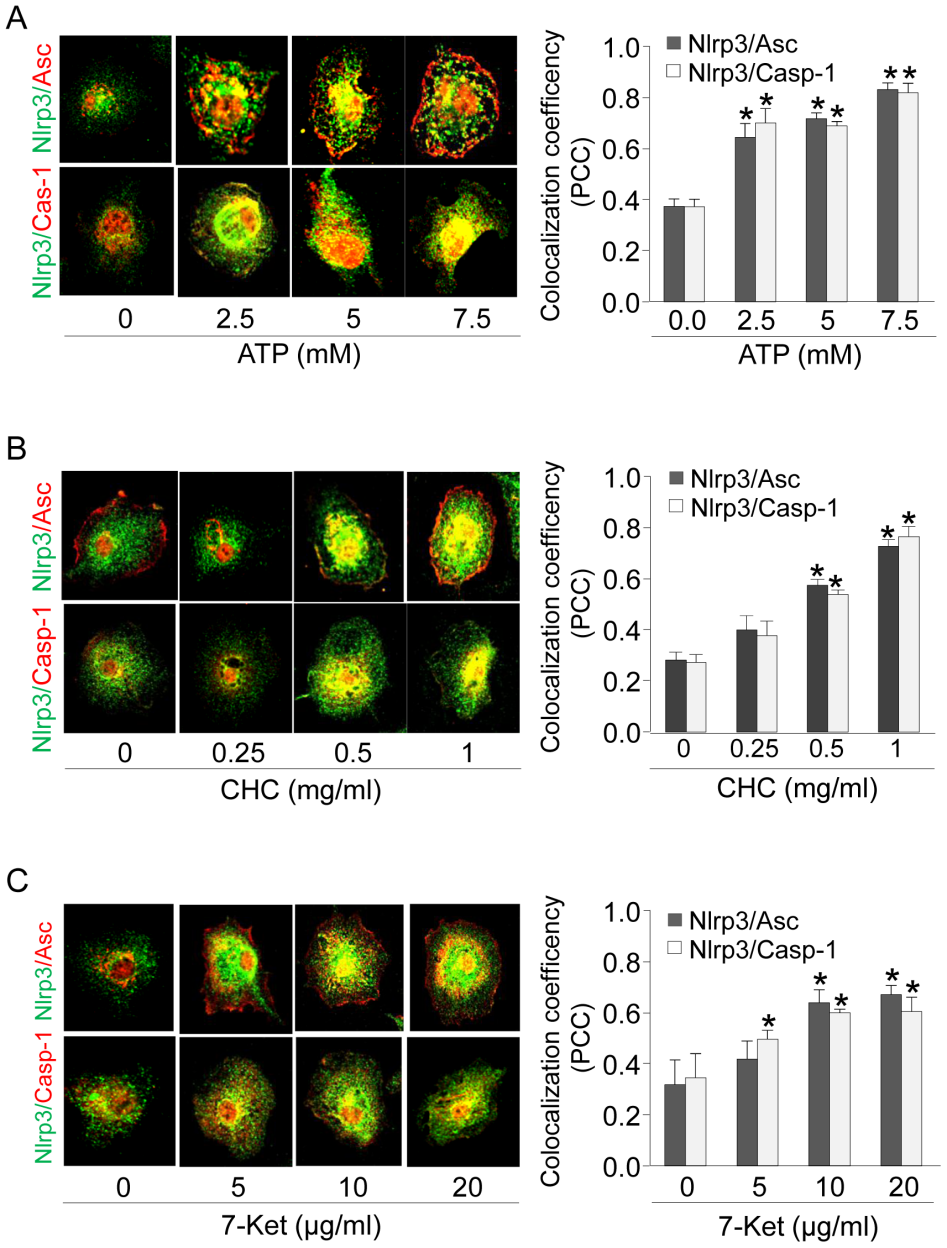
ATP, cholesterol crystal or 7-ketocholesterol increased inflammasome formation in Asc^+/+^ BMMs. (A) Representative confocal fluorescence images and summarized data depicting the effect of ATP (0–7.5 mM, 16 h) on the colocalization between Nlrp3 and Asc or caspase-1 in Asc^+/+^ BMMs. (B) Representative confocal fluorescence images and summarized data depicting the effect of cholesterol crystal (0–1 mg/ml, 16 h) on the colocalization between Nlrp3 and Asc or caspase-1 in Asc^+/+^ BMMs. (C) Representative confocal fluorescence images and summarized data depicting the effect of 7-ketocholesterol (0–20 µg/ml, 16 h) on the colocalization between Nlrp3 and Asc or caspase-1 in Asc^+/+^ BMMs. * P<0.05 vs. untreated control group (n = 6).

First, we examined the effects of non-atherogenic stimulus (ATP 0-7.5 mM) on the formation of Nlrp3 inflammasomes in cultured mouse BMMs. To this end, we determined whether Nlrp3, Asc and caspase-1 co-localize in the cells and form a complex after treatment with ATP. The confocal microscopy results (Fig. 1A) reveal a clear co-localization of Nlrp3 with Asc or Nlrp3 with caspase-1 upon ATP stimulation for 16 h in a dose-dependent manner, while almost no co-localization could be detected in untreated cells. This data indicates the aggregation and assembly of these inflammasome molecules after ATP-treatment. The Pearson correlation coefficient (PCC) represents co-localization efficiency of Nlrp3 with Asc or Nlrp3 with caspase-1 is also summarized. Significant increases in PCC occurred even at an ATP concentration of 2.5 mM. The co-localization of Nlrp3 with other inflammasome molecules after ATP-treatment suggests increased formation of Nlrp3 inflammasome in BMMs. ATP at higher concentration (5 and 7.5 mM) induced morphologic signs of cyotoxicity in BMMs. Thus, an ATP concentration of 2.5 mM was chosen for the rest of the experiments in the present study. In additional experiments, we also detected similar increases in Nlrp3 inflammasomes and colocalization pattern upon treatment of BMMs with atherogenic stimuli cholesterol crystals (CHC, 0-1 mg/ml) and 7-ketocholesterol (7-Ket, 0–20 µg/ml) (Fig.1B and 1C). We found that cholesterol crystals (0.5 mg/ml) and 7-ketocholesterol (10 µg/ml) optimally induced formation of Nlrp3 inflammasomes in BMMs with minimum cytotoxicity.

**Figure 2:**
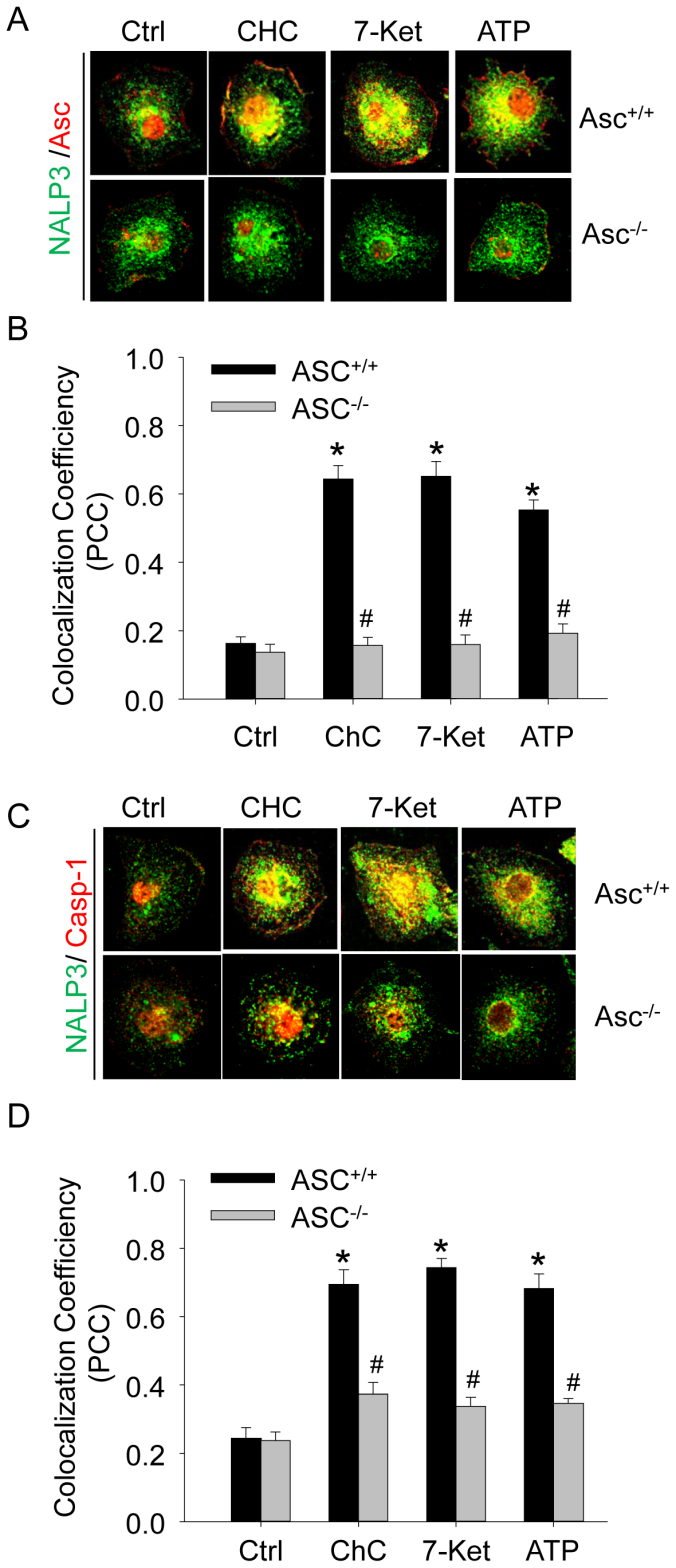
Effects of ATP, cholesterol crystals and 7-Ketocholesterol on Nlrp3 inflammasome formation in Asc^+/+^ and Asc^−/−^ BMMs. (A) Representative confocal fluorescence images depicting the effects of cholesterol crystals (CHC, 0.5 mg/ml, 16 h), 7-ketocholesterol (7-Ket, 10 µg/ml, 16 h), or ATP (2.5 mM, 16 h) on the colocalization between Nlrp3 and Asc from Asc^+/+^ and Asc^−/−^ BMMs. (B) Summarized data showing colocalization efficiency between Nlrp3 and Asc. (C) Representative confocal fluorescence images depicting the effects of cholesterol crystals (CHC, 0.5 mg/ml, 16 h), 7-ketocholesterol (7-Ket, 10 µg/ml, 16 h), or ATP (2.5 mM, 16 h) on the colocalization between Nlrp3 and caspase-1 from Asc^+/+^ and Asc^−/−^ BMMs. (D) Summarized data showing colocalization efficiency between Nlrp3 and caspase-1. * P<0.05 *vs*. untreated Asc^+/+^ control group; # P<0.05 Asc^−/−^ *vs*. Asc^+/+^ group (n = 6).

**Figure 3:**
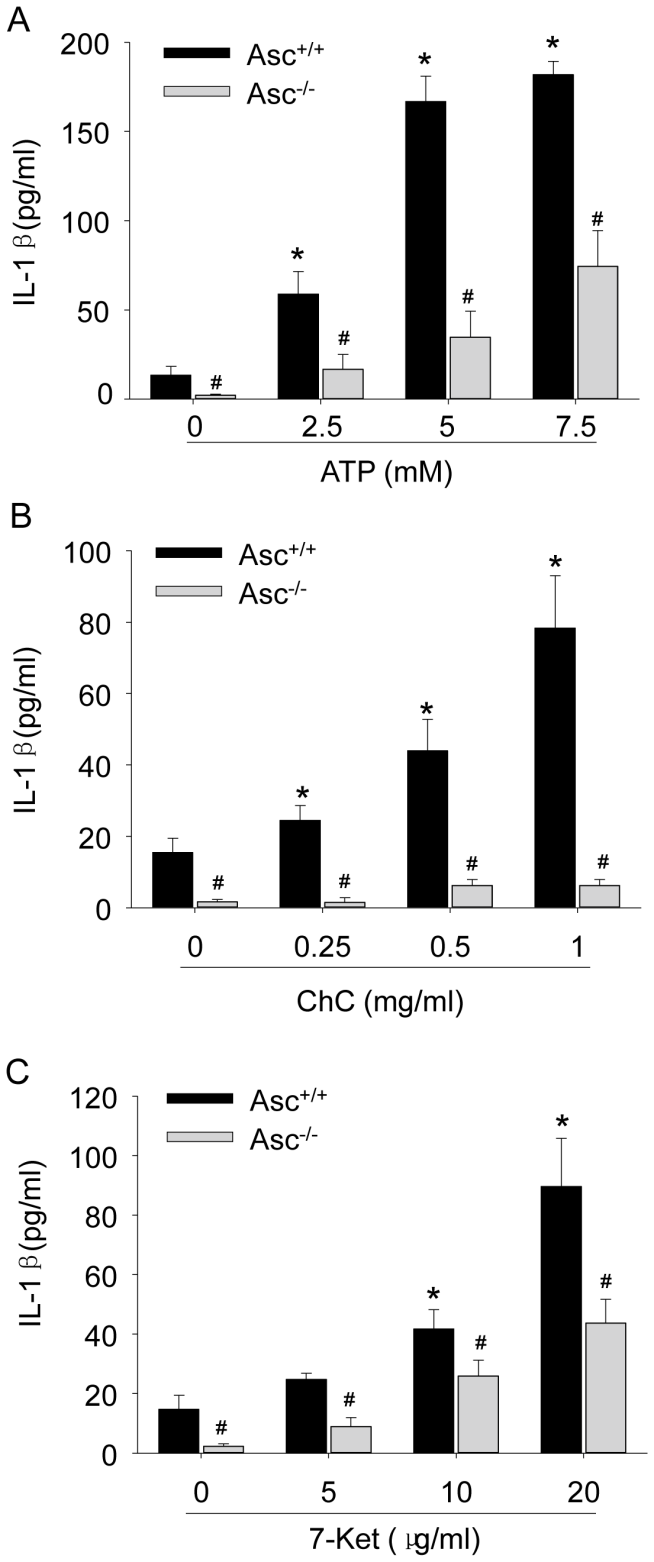
Effects of ATP, cholesterol crystals and 7-Ketocholesterol on IL-1β production in Asc^+/+^ and Asc^−/−^ BMMs. BMMs were stimulated with ATP (0–7.5 mM) (A), cholesterol crystals (CHC, 0–1 mg/ml) (B) or 7-ketocholesterol (7-Ket, 0–20 µg/ml) (C) for 16 h and IL-1β concentrations in the supernatants were determined by ELISA. * P<0.05 *vs*. untreated Asc^+/+^ control group; # P<0.05 Asc^−/−^ *vs*. Asc^+/+^ group (n = 6).

We further demonstrated that Asc gene deletion prevented the formation and activation of Nlrp3 inflammasomes induced by optimal dose of ATP (2.5 mM), cholesterol crystals (0.5 mg/ml) or 7-ketocholesterol (10 µg/ml) in BMMs (Fig.2A–D). In addition, ATP, cholesterol crystal or 7-ketocholesterol significantly increased the production of inflammasome product IL-1β in Asc^+/+^ BMMs, however, such increases were abolished in Asc^−/−^ BMMs (Fig.3A–C).

### Asc is essential for ATP-induced caspase-1 activation in BMMs

**Figure 4:**
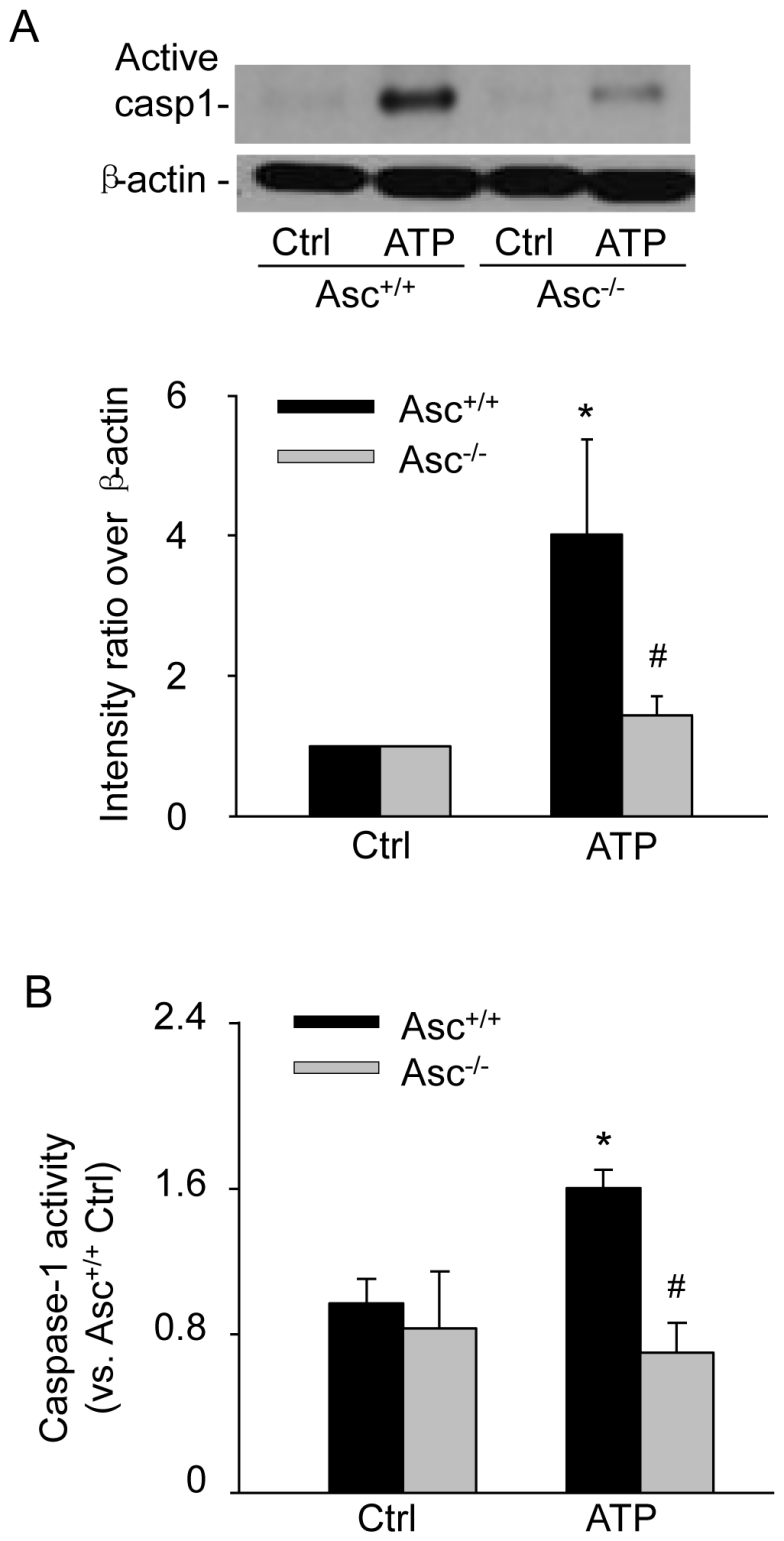
Asc gene knockout inhibited ATP-induced caspase-1 activation in BMMs. (A) Western blot analysis showing the effect of ATP (2.5 mM, 16 h) on active caspase-1 expression and the quantitative analysis (lower blot). (B) Summarized data of FLICA assays quantifying the relative caspase-1 activity compared to control. * P<0.05 *vs*. untreated Asc^+/+^ control group; # P<0.05 *vs*. Asc^+/+^ ATP group (n = 6).

To further confirm that whether ATP induced caspase-1 activation following inflammasome assembly, we measured the cleavage of pro-caspase-1 into active caspase-1 by Western blot analysis and analyzed caspase-1 activity using specific FLICA probes by flow cytometry. As shown in Fig. 4A, ATP at 2.5 mM significantly increased the formation of active caspase-1 (20 kDa), the cleaved fraction from pro-capaspase-1. Consistently, ATP (2.5 mM)-induced increases in caspase-1 activity were substantially attenuated in Asc^−/−^ BMMs compared to those in Asc^+/+^ BMMs (Fig.4B).

### Activation of Nlrp3 inflammasomes increases lipid accumulation in BMMs

**Figure 5:**
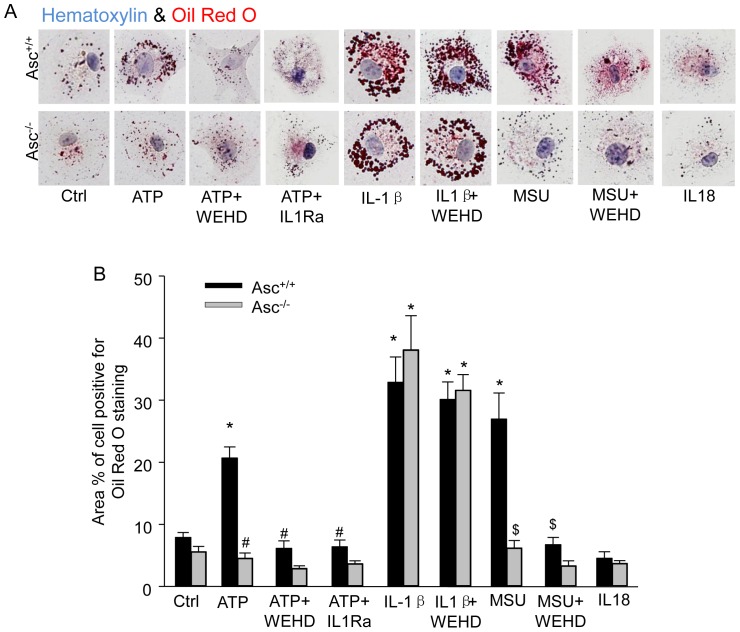
Increased lipid accumulation in BMMs with activation of Nlrp3 inflammasomes by ATP. BMMs were primed with LPS (1 ng/ml) for 3 h and treated with ATP (2.5 mM, 16 h), IL1β (0.5 ng/ml), MSU (100 µg/ml), or IL18 (25 nM). Some group of BMMs were treated with ATP or MSU in the presence of caspase-1 inhibitor WEHD (0.15 µg/ml) or IL1R antagonist (IL1Ra, 40 ng/ml). Then, BMMs were loaded with oxLDL (10 µg/mL) for 16 hours. (A) Light microscopic images show oil red O stained BMMs. Cells were counterstatined with hematoxylin. (B) Summarized data showing area percentage of each cell positive for Oil red O staining in BMMs. * P<0.05 *vs*. untreated Asc^+/+^ control group; # P<0.05 *vs*. Asc^+/+^ ATP group; $ P<0.05 vs. Asc^+/+^ MSU group (n = 6).

Next, we examined whether activated Nlrp3 inflammasomes by other non-atherogenic stimuli induce alterations in macrophages that may contribute in the concert with other factors to atherogenesis. High concentration of intercellular ATP was generally considered as a danger signal; however, ATP alone is non-atherogenic. Thus, we examined whether the activation of the inflammasome by non-atherogenic stimulus ATP may result in dysregulation of lipid metabolism and deposition in BMMs, a change that occurs early in macrophages involved in the development of atherosclerosis. As shown in Figure 5A and summarized data in Figure 5B, ATP significantly increased lipid loading in BMMs as detected by Oil Red O staining. The caspase-1 inhibitor Z-WEHD-FMK (WEHD), IL1R antagonist (IL1Ra) or Asc gene deletion blocked the lipid loading of BMMs by ATP. Similarly, another non-lipid inflammasome stimulus MSU also enhanced lipid deposition which was blocked by WEHD. Moreover, administration of Nlrp3 inflammasome product, IL-1β but not IL18, produced more dramatic increase in lipid accumulation, which was not blocked by WEHD or Asc gene deletion. These results suggest that inflammasome activation in macrophages stimulated by non-atherogenic stimuli such as ATP also increases their lipid loading potential and sensitivity to form foam cells.

### Asc gene deletion and capase-1 inhibition block inflammasome-induced cholesterol deposition in lysosomes of BMMs

**Figure 6:**
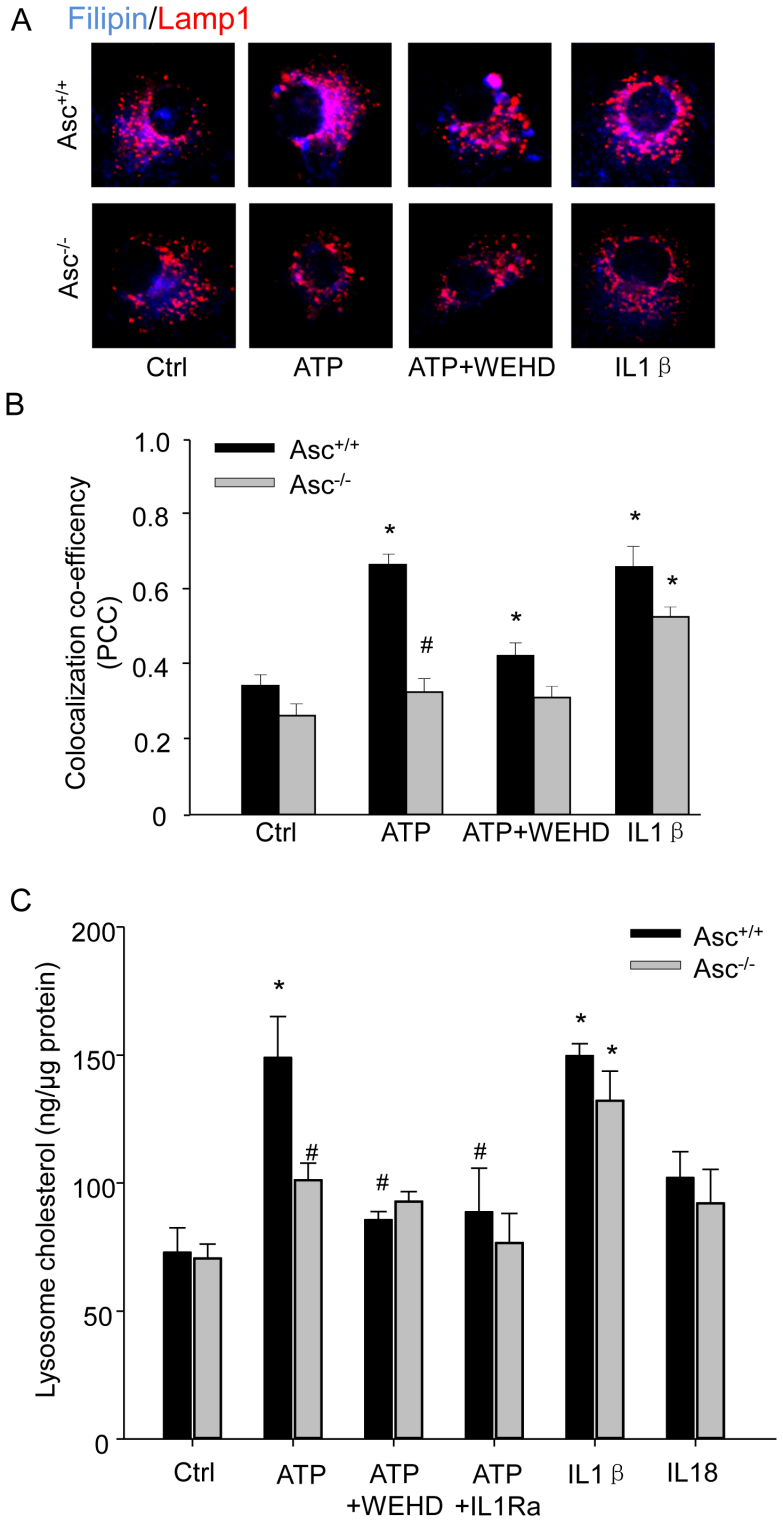
Asc gene deletion and caspase-1 inhibition blocked cholesterol deposition in lysosomes. BMMs were primed with LPS (1 ng/ml) for 3 h and treated with ATP (2.5 mM, 16 h) in the absence or presence of caspase-1 inhibitor WEHD (0.15 µg/ml), or IL1β (0.5 ng/ml) alone. Then BMMs were loaded with oxLDL (10 µg/mL) for 16 hours. (A) Representative confocal fluorescent images showing the colocalization between filipin (cholesterol) and Lamp1 (lysosome marker). An increase in the purple color in overlay images indicates increased cholesterol trapping in lysosomes. (B) Quantified and summarized data showing co-localization co-efficiency between filipin and Lamp1. (C) Effect of Asc gene deletion on cholesterol level in isolated lysosomes from BMMs. Lysosomes were isolated from Asc^+/+^ and Asc^−/−^ BMMs and cholesterol concentration in these isolated lysosomes were determined using a standard fluorescence assay kit. Some groups of cells were pretreated with ATP in the presence of IL1R antagonist (IL1Ra, 40 ng/ml) or IL18 (25 nM) alone. * P<0.05 *vs*. untreated Asc^+/+^ control group; # P<0.05 *vs*. Asc^+/+^ ATP group (n = 6).

To gain insight into the composition of the lipids deposited in macrophages, we performed cholesterol staining with filipin. As shown in Fig. 6A and 6B, intense and widespread filipin staining (purple color) was found in BMMs stimulated by ATP indicating accumulation of cholesterol in the lysosomes of these cells. ATP-induced cholesterol deposition in lysosomes was blocked by WEHD and it was absent in Asc^−/−^ BMMs (Fig. 6A and 6B). Vice versa, IL-1β induced lysosomal deposition of cholesterol in both Asc^+/+^ and Asc^−/−^ BMMs (Fig.6A and 6B). Further evidence for the role of inflammasome in lipid accumulation in the lysosomes was obtained by measuring cholesterol in isolated lysosomes. These studies confirmed the filipin stainings and show that ATP increased lysosomal cholesterol concentration, which was markedly attenuated by WEHD or Asc gene deletion, while increased by IL-1β (Fig.6C). Further, ATP-induced increases in lysosomal cholesterol level was blocked by IL1R antagonist and IL18 had no effect on lysosomal cholesterol level. These results again confirm that non-atherogenic stimulation-induced Nlrp3 inflammasome activation and its product IL-1β impair the ability of macrophages for clearance of cholesterol in lysosomes.

### Activation of inflammasome impairs intracellular lipid trafficking

**Figure 7:**
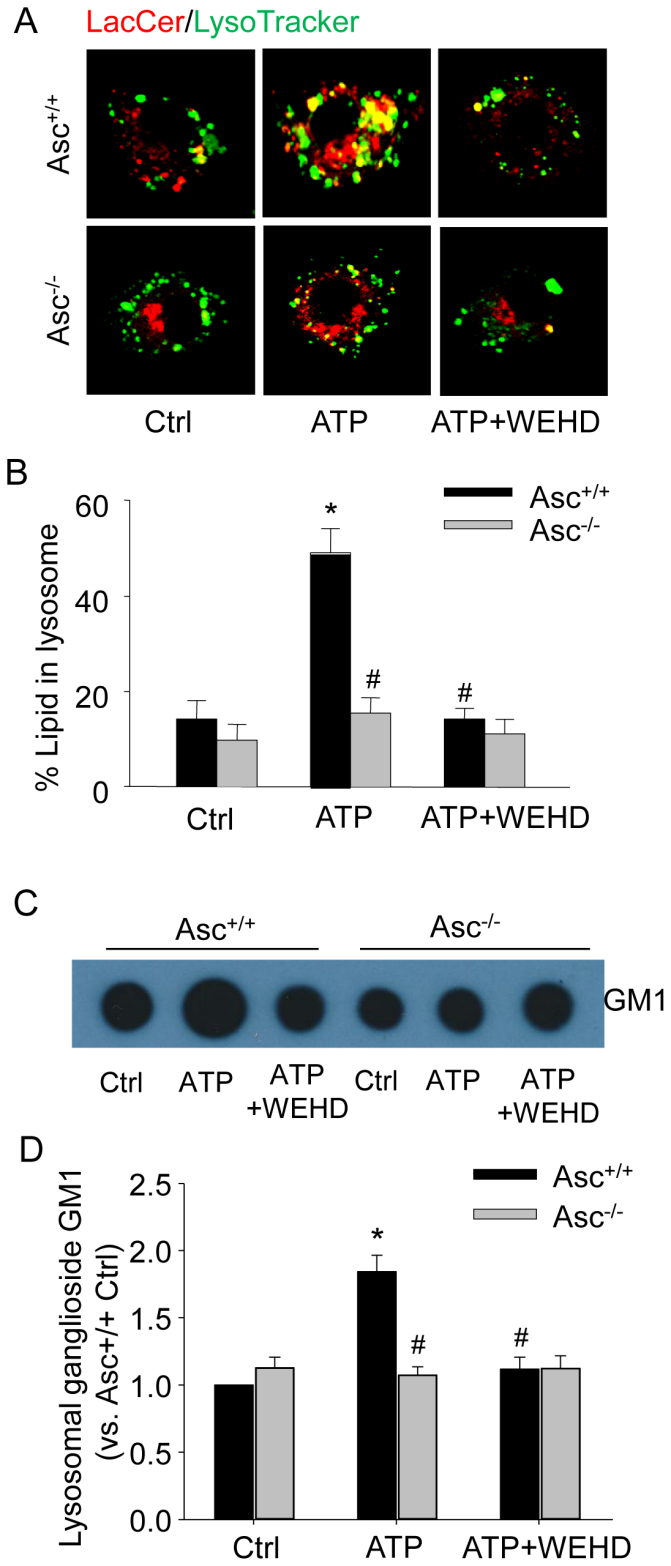
ATP-induced lipid trafficking in lysosomes was blocked in Asc^−/−^ BMMs. Asc^+/+^ and Asc^−/−^ BMMs were primed with low dose of LPS (1 ng/ml) for 3 h and treated with ATP (2.5 mM, 16 h) in the absence or presence of caspase-1 inhibitor WEHD (0.15 µg/ml). (A) BMMs were incubated with BSA-conjugated BODIPY FL-C_5_-lactosylceramide (LacCer) and lipid trafficking was tested by following LacCer trafficking. Representative confocal fluorescent images show the co-localization between LacCer (red color) and Lysotracker (green color). An increase in the yellow color in overlay images indicates increased LacCer trafficking to the lysosomes. (B) Quantified and summarized data showing the percent of lipid in lysosomes. (C) The ganglioside GM1 levels in isolated lysosomes from BMMs were determined by dot blot analysis. Represent dot blot image shows the ganglioside GM1 level in isolate lysosome homogenates as detected by cholera toxin-conjugated HRP. (D) Summarized analysis of ganglioside GM1 in lysosomes. * P<0.05 *vs*. untreated Asc^+/+^ control group; # P<0.05 *vs*. Asc^+/+^ ATP group (n = 6).

To study the role of inflammasome in lipid trafficking in living cells, we loaded BMMs with BODIPY FL-C_5_-LacCer and observed its intracellular trafficking using confocal microscopy. After endocytosis into vesicles, this fluorescent ceramide analog is trafficked to intracellular organelles such as Golgi apparatus or lysosomes. Non-atherogenic stimulation of BMMs by ATP significantly increased the overlap (yellow punctuated staining) between LacCer (red) and LysoTracker (green) indicating that LacCer probes were accumulated in the lysosomes (Fig. 7A and 7B). Such accumulation of LacCer in lysosomes was substantially attenuated by WEHD or Asc gene deletion (Fig. 7A and 7B). To examine whether ATP results in lysosomal accumulation of sphingolipids, we directly measured the lysosomal content of a typical glycosphingolipid ganglioside GM1 in BMMs. As shown in Fig.7C and 7D, ATP markedly increased ganglioside GM1 contents in lysosomes, which was attenuated by ASC gene deletion or caspase-1 inhibitor WEHD.

### Enhanced macrophage migration by activation of inflammasomes is independent of IL-1β

**Figure 8:**
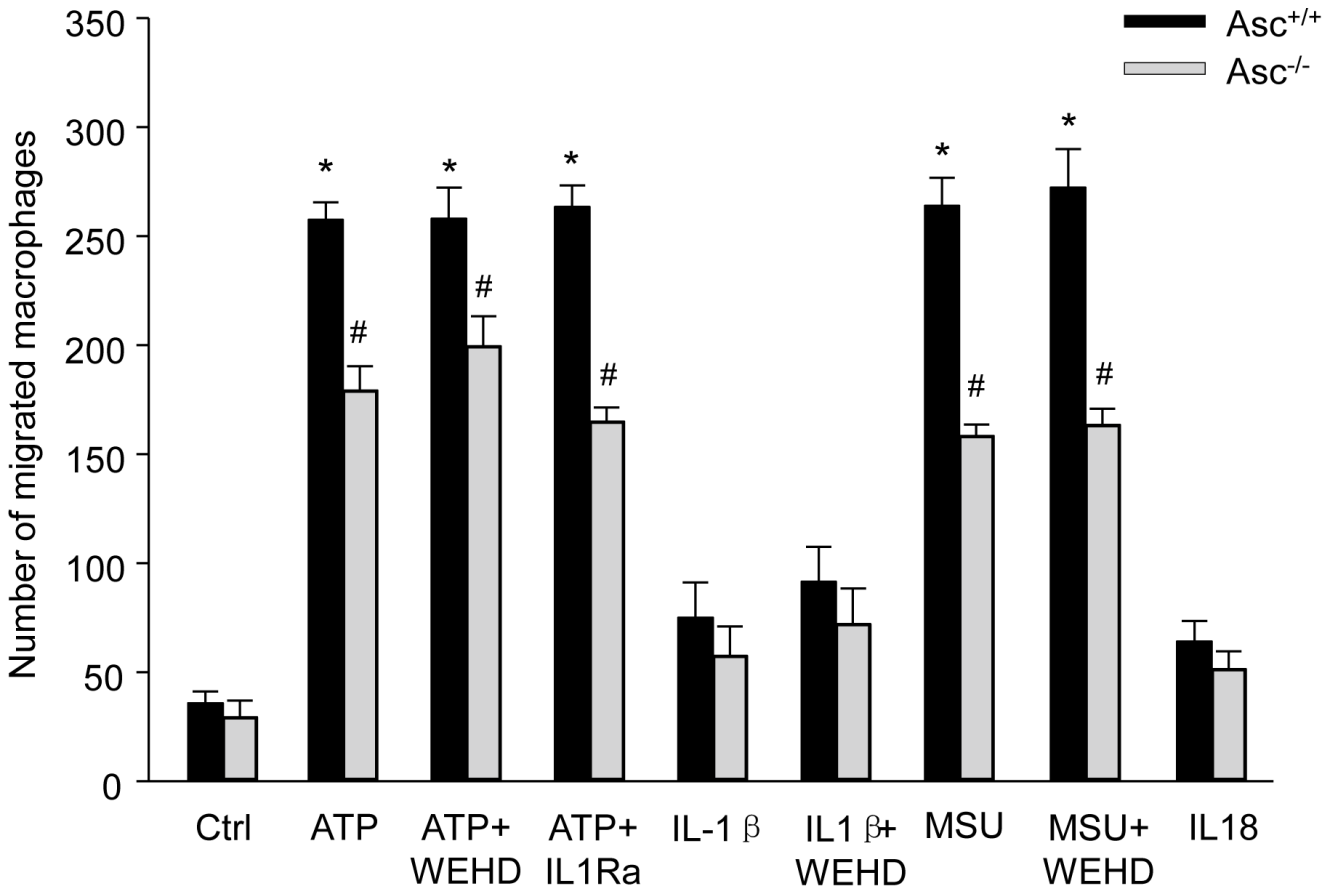
Effect of inflammasome activation on macrophage migration *in vitro*. Macrophage migration *in vitro* was assayed using Transwell inserts with a 5 µm porous membrane. The migratory cells on the lower side of insert membrane were quantified. BMMs in Transwell inserts were primed with LPS (1 ng/ml) for 3 h and treated with ATP (2.5 mM, 16 h), IL1β (0.5 ng/ml), MSU (100 µg/ml), or IL18 (25 nM) in the absence or presence of caspase-1 inhibitor WEHD (0.15 µg/ml) or IL1R antagonist (IL1Ra, 40 ng/ml). Quantification of the transwell assays reveals the ability of BMMs to migrate from inside Transwell inserts through the membrane upon stimulation. * P<0.05 *vs*. untreated Asc^+/+^ control group; # P<0.05 Asc^−/−^ *vs.* Asc^+/+^ group (n = 6).

We next examined macrophage migration *in vitro* in a Boyden chamber assay using Transwell inserts with a 5 µm porous membrane. Cells that migrated on the lower side of the insert membrane were quantified. Enhanced migration was observed in Asc^+/+^ BMMs when they were incubated with non-atherogenic ATP (Fig. 8), which was attenuated in Asc^−/−^ BMMs. However, macrophage migration induced by such non-atherogenic stimulation was not blocked by either WEHD or IL1R antagonist. Similarly, MSU markedly increased migration of BMMs which was attenuated by Asc gene deletion but was not affected by WEHD. Further, ATP-induced migration of BMMs was not mimicked by either IL-1β or IL18. These results suggest that macrophage migration enhanced by Nlrp3 inflammasome activation is independent of IL-1β and IL18.

### Asc gene deletion inhibits macrophage migration in vivo

**Figure 9:**
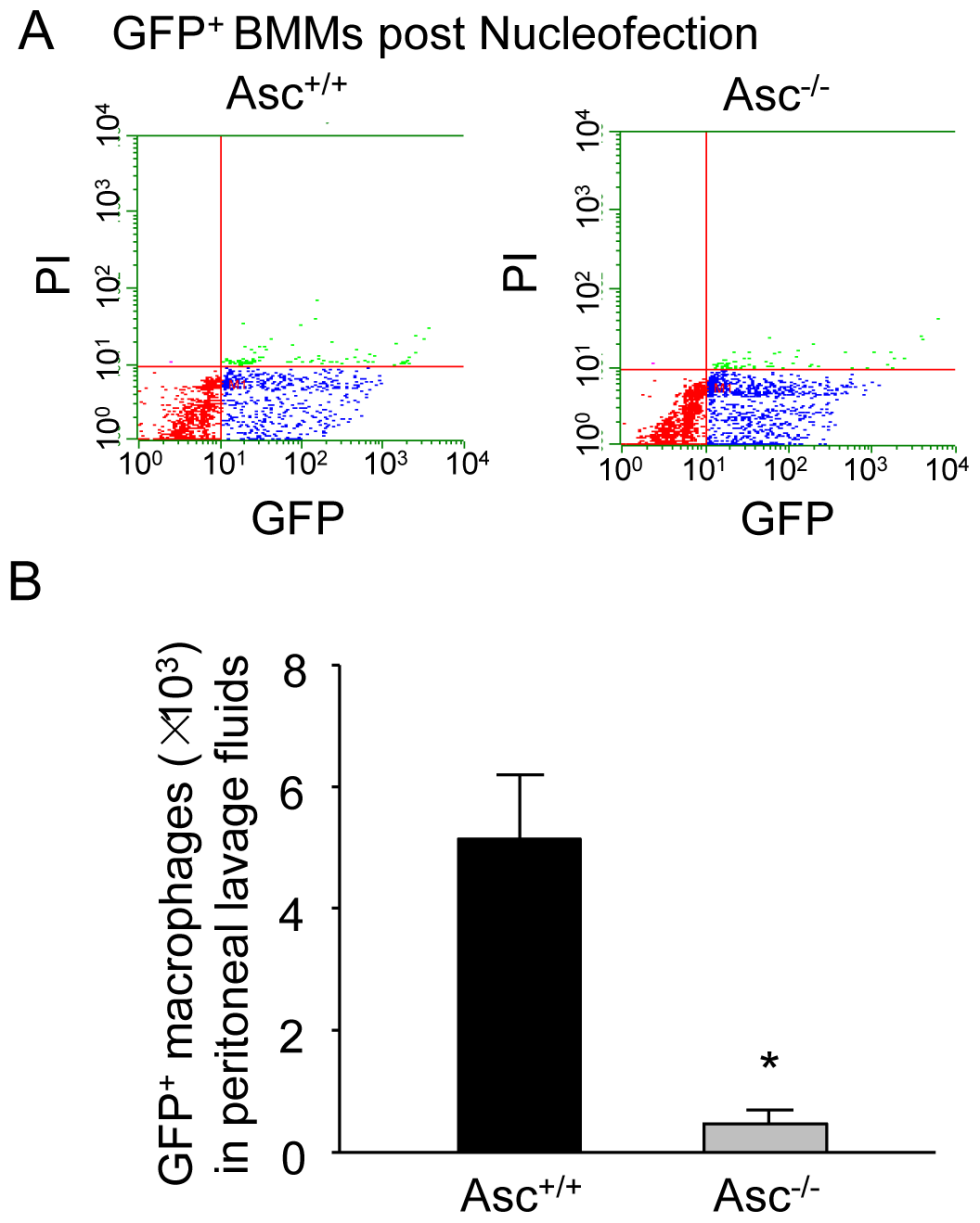
Macrophage migration *in vivo* is attenuated by Asc-deficiency. (A) GFP-expressing Asc^+/+^ and Asc^−/−^ BMMs were obtained by Nucleofection of BMMs with GFP-encoding plasmids. Representative dot plots from flow cytometry analysis show the GFP expression and cell viability (PI) in Asc^+/+^ and Asc^−/−^ BMMs post Nucleofection with GFP plasmids. (B) GFP-expressing Asc^+/+^ or Asc^−/−^ BMMs were intravenously injected in Asc^+/+^ mice with Zymosan A-induced peritonitis. The numbers of GFP^+^ macrophages in peritoneal lavage fluids of Asc^+/+^ mice were quantified and compared. * P<0.05 *vs*. Asc^+/+^ BMMs (n = 4).

To evaluate the effect of Asc gene deletion on macrophage migration *in vivo*, we measured Zymosan A-induced recruitment of macrophages into peritoneal cavity using a murine peritonitis model and GFP-expressing BMMs. To obtain GFP-expressing BMMs, we used traditional liposome method to transfect BMMs with GFP cDNA plasmids. However, the transfection efficiency was very low (∼2%) (data not shown), which may be due to hard-to-transfect nature of primary cells including BMMs. To obtain high transfection efficiency as well as cell viability, the present study introduced GFP gene into Asc^+/+^ and Asc^−/−^ BMMs by a novel Nucleofection technology that directly delivers GFP cDNA plasmids into nucleus ensuring GFP expression. Indeed, as shown in Fig.9A, Nucleofection method had little effects on cell viability for both Asc^+/+^ and Asc^−/−^ BMMs (PI^−^ cells >95%) but markedly increased the transfection efficiency as shown by increased GFP^+^PI^−^ BMM population (GFP^+^PI^−^ cells were quantified as live BMMs expressing GFP) in both Asc^+/+^ BMMs (41% of total cells were GFP^+^PI^−^ cells) and Asc^−/−^ BMMs (37% of total cells were GFP^+^PI^−^ cells). For *in vivo* migration assay, we intravenously injected these GFP-expressing Asc^+/+^ or Asc^−/−^ BMMs into Asc^+/+^ mice (*i.v.* 10^5^ GFP expressing BMMs per mouse). Then, 2 h after intraperitoneal injection of Zymosan A, we isolated the peritoneal lavage fluids, collected peritoneal cells and analyzed these cells by flow cytometry as described in the Methods. As shown in Fig. 9B, when Asc^+/+^ mice were intravenously injected with 10^5^ GFP-expressing Asc^+/+^ BMMs, approximately 5×10^3^ of these GFP-expressing cells were detected in the peritoneal fluid of each mouse after Zymosan A injection. In contrast, when Asc^+/+^ mice were injected with 10^5^ GFP-expressing Asc^−/−^ BMMs, much less GFP-expressing Asc^−/−^ BMMs (∼500 cells) were detected in the peritoneal lavage fluids after mice were injected with Zymosan A. Thus, these data suggest that Asc is needed for macrophage migration *in vivo*.

## Discussion

The present study demonstrated that formation and activation of Nlrp3 inflammasomes induced by the non-atherogenic danger signal ATP was associated with increased lipid deposition in lysosomes and enhanced migration ability in macrophages. In contrast, Asc gene deletion markedly abolished Nlrp3 inflammasome activation, attenuated lysosomal lipid deposition and decreased macrophage migration ability. These results suggest that the formation and activation of Nlrp3 inflammasomes by non-atherogenic stimulation alters macrophage function and increase the susceptibility of these cells to formation of foam cells.

Recent studies have suggested that atherogenesis can be initiated by endogenous molecules mediating sterile inflammation. One such molecule is crystalline cholesterol, as engulfment of cholesterol crystals by macrophages leads to the activation and recruitment of inflammatory cells, endothelial dysfunction and plaque formation. Nlrp3 acts as the sensory component to recognize these endogenous danger signals [9], [31], [32], when Asc and caspase-1 are recruited to form a protein complex, where caspase-1 is activated. The present study aimed to explore whether Nlrp3 inflammasome activation by non-atherogenic stimuli contributes to the initial steps of atherogenesis by altering macrophage function. By confocal microscopic and biochemical analyses, we demonstrated that in macrophages, Asc is essential for Nlrp3 inflammasome formation and activation induced by the prototype, non-atherogenic stimulus ATP as well as by typical atherogenic stimulus 7-ketocholesterol and a recently identified atherogenic stimulus cholesterol crystal. The present study was designed to examine whether activation of inflammasomes may also cause macrophage dysfunction in addition to turning on the classical inflammatory responses. In this regard, we next demonstrated that Nlrp3-mediated inflammasome activation caused macrophage dysfunction including increased lipid deposition and enhanced migration. Our results support the view that Nlrp3 inflammasome activation not only instigates inflammatory response, but also has direct effects to alter cell functions leading to cell injury.

The present study provides the first evidence that Nlrp3 inflammasome activation increased cholesterol deposition in lysosomes of macrophages exposed to oxLDL. Asc gene deletion and caspase-1 inhibition by WEHD blocked ATP-induced abnormal lysosomal cholesterol deposition in these macrophages. Moreover, IL-1β alone strongly increased lipid deposition and lysosomal cholesterol accumulation implicating the role of caspase-1/IL1β in mediating such action of an inflammasome in macrophages. This result is consistent with previous studies showing that IL1β is involved in a variety of cellular activities, including cell proliferation, differentiation, and apoptosis in addition to function as an important mediator of the inflammatory response [1], [28], [33]. Taken together, our data suggest that Nlrp3 inflammasome activation facilitates transformation of macrophages into lipid-laden foam cells, which reveals a novel role of the Nlrp3 inflammasome in regulating macrophage function. Atherosclerosis is a disease characterized by accumulation of lipids and an inflammatory response in the arterial intima, resulting in the formation of plaque that can lead to arterial narrowing and that is susceptible to rupture with acute thrombotic occlusion. In the initial stage of atherogenic inflammation, monocyte-derived macrophages perform a critical role by internalizing oxLDL through scavenger receptors and emigrating from the inflammatory site after clearing the lipids [34], [35]. The release of high concentrations of ATP in the arterial wall associates with necrosis, which occurs only in advanced lesions. It should be noted that the present study used ATP as a prototype non-lipid inflammasome stimulus. By using another inflammasome stimulus MSU, we demonstrated that lipid deposition is associated with inflammasome activation rather than the forms of inflammasome stimuli. Thus, it is plausible that *in vivo* activation of the Nlrp3 inflammasome activation by non-lipid danger factors other than ATP increases macrophage transformation into lipid-laden foam cells, which remain in the lesion after clearing the lipids. Increased numbers of macrophages trapped in the arterial intima may provoke an inflammatory response at the local site, ultimately contributing to the initiation and development of atherosclerosis.

Our data suggest that abnormal lipid deposition and lysosomal cholesterol accumulation are due to impaired intracellular lipid trafficking in macrophages upon Nlrp3 inflammasome activation by non-atherogenic stimulus ATP. The relationship between cholesterol and glycosphingolipid homeostasis is complex. Previous studies have shown that cholesterol and glycosphingolipids have high affinity for one another and are the two main components of lipid raft microdomains, and therefore accumulation of these sphingolipids in lysosomes can cause lysosomal trapping and accumulation of the cholesterol [36]. In the present study, we found that inflammasome activation by ATP increased lysosomal LacCer accumulation in macrophages in the absence of cholesterol loading (i.e. these BMMs were not treated with oxLDL) (Fig.7A and 7B). These data suggest that inflammasome activation may impair the post-lysosomal trafficking of LacCer to the Golgi apparatus resulting in accumulation of LacCer in lysosomes. Consistently, ATP markedly increased lysosomal contents of glycosphingolipid such as ganglioside GM1, which were attenuated by ASC gene deletion or caspase-1 inhibitor WEHD (Fig.7C and 7D). Thus, our results support the view that Nlrp3 inflammasome activation impairs post-lysosomal trafficking of glycosphingolipids to the Golgi, resulting in lysosomal accumulation of these sphingolipids, which may lead to retention of cholesterol in the lysosomes.

In addition to modulate lipid trafficking and deposition in macrophages, our *in vitro* and *in vivo* data also reveal that inflammasome activation may increase macrophage migration ability. The data from our *in vitro* study show attenuated migration activity of Asc^−/−^ macrophages compared to Asc^+/+^ macrophages upon stimulation with non-atherogenic ATP. However, such increases in macrophage migration ability were not blocked by WEHD or mimicked by IL-1β. Thus, the mechanisms by which activated Nlrp3 inflammasomes enhance macrophage lipid deposition and migration may be different. It should be noticed that Asc deficiency only partially attenuated macrophage migration ability induced by ATP in our *in vitro* migration assays, which may be due to activation of Asc-independent mechanism by ATP that enhances macrophage migration ability. Previous studies have shown that P2Y2, a P2Y-family purinergic G-protein coupled receptor can be activated to induce THP-1 monocyte migration even at low ATP concentration (100 nM) [37]. In the present study, the BMMs was treated high ATP concentration (2.5 mM) which has been well documented to activate P2X7 receptor leading to Nlrp3/Asc/caspase-1 inflammasome complex formation. Thus, our data support the view that ATP has dual effects on macrophage migration ability. First, ATP initiates P2X7-mediated Nlrp3/Asc/caspase-1 signaling that regulates macrophage migration ability. In addition, ATP at high concentration may also activate inflammasome-independent P2Y2 receptor-mediated chemotactic responses of monocytes/macrophages.

Our findings from animal experiments further confirmed the non-inflammatory role of Asc in macrophage migration *in vivo*. The *in vivo* macrophage peritoneal influx model used in these studies is an appropriate system to probe the non-inflammatory effects of Asc gene deletion on macrophage migration to the inflammatory lesions. In this system, only wild-type mice were used for peritonitis model and all mice were injected with same number of GFP-expressing Asc^+/+^ or Asc^−/−^ BMMs in the circulation. Thus, the Zymosan A-induced inflammatory responses (such as chemokine production) in host mice receiving either GFP-expressing Asc^+/+^ or Asc^−/−^ BMMs are similar and the factors influencing monocyte recruitment are eliminated. To this respect, the difference of migration ability between injected Asc^+/+^ or Asc^−/−^ BMMs would be attributed to Asc-dependent non-inflammatory mechanisms in these cells. Indeed, abrogation of Asc gene markedly blocked migration ability of macrophages in our *in vivo* animal experiments. In the *in vivo* model, Nlrp3 inflammasome and Asc-dependent migration of macrophages can be activated by multiple mechanisms such as reactive oxygen species (ROS) or lysosome destabilization-cathepsin B cascade in addition to ATP/P2X7 signaling [38]. This may explain that the reduction of migration in Asc^−/−^ BMMs was more pronounced in *in vivo* model compared with that in *in vitro* assays using ATP. The mechanism for Asc inflammasome-dependent migration in macrophages is unknown. Recent studies suggest that Asc may play a role in regulating macrophage migration ability via Rac signaling pathway [39]. Asc controls mRNA stability and expression of DOCK2, a guanine nucleotide exchange factor that mediates Rac-dependent signaling in immune cells and Asc deficiency results in impaired Rac-mediated actin polymerization leading to defective lymphocyte migration in Asc^−/−^ mice [39]. It is possible that activation of Asc-associated inflammasome in macrophages plays a similar role in regulating macrophage migration ability.

In summary, the present study revealed that activation of inflammasomes even induced by non-lipid stimuli may lead to abnormal lipid metabolism in macrophages and thereby increases the potential to accumulate cholesterol in lysosomes as well as regulates migration of macrophages. These non-inflammatory effects of inflammasomes on macrophages may ultimately result in formation of foam cells promoting atherosclerosis.
